# Garcinol Exhibits Anti-Neoplastic Effects by Targeting Diverse Oncogenic Factors in Tumor Cells

**DOI:** 10.3390/biomedicines8050103

**Published:** 2020-04-30

**Authors:** Vaishali Aggarwal, Hardeep Singh Tuli, Jagjit Kaur, Diwakar Aggarwal, Gaurav Parashar, Nidarshana Chaturvedi Parashar, Samruddhi Kulkarni, Ginpreet Kaur, Katrin Sak, Manoj Kumar, Kwang Seok Ahn

**Affiliations:** 1 Department of Histopathology, Postgraduate Institute of Medical Education and Research (PGIMER), Chandigarh, Punjab 160012, India; vaishali.pgi@gmail.com; 2 Department of Biotechnology, Maharishi Markandeshwar (Deemed to be University), Mullana-Ambala, Haryana 133207, India; diwakaraggarwal@yahoo.co.in (D.A.); or parashar_panchal@yahoo.co.in (G.P.); nidarshanachaturvedi@gmail.com (N.C.P.); 3 Graduate School of Biomedical Engineering, ARC Centre of Excellence in Nanoscale Biophotonics (CNBP), Faculty of Engineering, The University of New South Wales, Sydney 2052, Australia; 1990jagjit@gmail.com; 4 Shobhaben Pratapbhai Patel School of Pharmacy and Technology Management, SVKM’S NMIMS, Vileparle-West, Mumbai 400056, India; sami.kul30@gmail.com (S.K.); ginpreet.aneja@gmail.com (G.K.); 5 NGO Praeventio, Tartu 50407, Estonia; katrin.sak.001@mail.ee; 6 Department of Chemistry, Maharishi Markandeshwar University, Sadopur 133001, India; manojraju27@gmail.com; 7 Department of Science in Korean Medicine, College of Korean Medicine, Kyung Hee University, 24 Kyungheedae-ro, Dongdaemun-gu, Seoul 02447, Korea

**Keywords:** garcinol, apoptosis, cell cycle, anti-angiogenesis, anti-inflammation

## Abstract

Garcinol, a polyisoprenylated benzophenone, is the medicinal component obtained from fruits and leaves of *Garcinia indica* (*G. indica*) and has traditionally been extensively used for its antioxidant and anti-inflammatory properties. In addition, it has been also been experimentally illustrated to elicit anti-cancer properties. Several in vitro and in vivo studies have illustrated the potential therapeutic efficiency of garcinol in management of different malignancies. It mainly acts as an inhibitor of cellular processes via regulation of transcription factors NF-κB and JAK/STAT3 in tumor cells and have been demonstrated to effectively inhibit growth of malignant cell population. Numerous studies have highlighted the anti-neoplastic potential of garcinol in different oncological transformations including colon cancer, breast cancer, prostate cancer, head and neck cancer, hepatocellular carcinoma, etc. However, use of garcinol is still in its pre-clinical stage and this is mainly attributed to the limitations of conclusive evaluation of pharmacological parameters. This necessitates evaluation of garcinol pharmacokinetics to precisely identify an appropriate dose and route of administration, tolerability, and potency under physiological conditions along with characterization of a therapeutic index. Hence, the research is presently ongoing in the dimension of exploring the precise metabolic mechanism of garcinol. Despite various lacunae, garcinol has presented with promising anti-cancer effects. Hence, this review is motivated by the constantly emerging and promising positive anti-cancerous effects of garcinol. This review is the first effort to summarize the mechanism of action of garcinol in modulation of anti-cancer effect via regulation of different cellular processes.

## 1. Introduction

Despite the continuous advancements in diagnostic and therapeutic methods, cancer has still remained one of the most dreadful diseases in 21st century all over the world, with 18.1 million new cases and 9.6 million mortalities estimated by 2018 [1]. In the past few decades, chemoprevention, i.e., the use of natural compounds to retard or reverse the carcinogenic processes, has become a promising strategy to decrease the formation and progression of malignant disorders. Many phytochemicals present in the human diet have identified as potential chemopreventive and/or chemotherapeutic substances [2,3,4,5,6,7]. Moreover, nutrition and diet have been associated with about one third of all the cancer cases [8], whereas consumption of dietary substances present in vegetables, fruits, and medicinal herbs can reduce the development of cancerous processes. Therefore, searching for novel natural compounds and identifying their cellular action mechanisms for chemoprevention have become a fascinating research area.

Garcinol is one of such important phytochemicals with several anticancer properties. Garcinol belongs to the group of polyisoprenylated benzophenones obtained from the fruits and leaves of diverse *Garcinia* species including *G. indica*, a small evergreen tree found in the tropical Asia and Africa [9,10,11]. This phytochemical has been shown to reveal potential therapeutic effects both in vitro and in vivo studies, including antioxidant, anti-inflammatory and anticancer activities [11]. The effects of garcinol on induction of cell cycle arrest and/or apoptotic death and the prevention of angiogenic and metastatic events have been described in numerous reports performed with various tumor cell lines and animal models, suggesting that this phytochemical agent might be able to control malignant development at different stages [11,12,13,14,15,16,17,18]. Garcinol can act as an efficient inhibitor for diverse cellular pathways closely related to carcinogenic processes, such as extracellular signal regulated kinases 1/2 (ERK1/2), phosphoinositide 3-kinase (PI3K)/AKT serine/threonine kinase (Akt), wingless (Wnt)/β-catenin, signal transducer and activator of transcription 3 (STAT3), and/or nuclear factor kappa B (NF-κB) signaling [19]. Furthermore, recent studies have also shown that the administration of tumoral cells with conventional cytotoxic chemotherapeutic drugs, such as cisplatin or paclitaxel, in the presence of garcinol can lead to a significant increase in the treatment outcome of traditional anticancer agents [10,20,21].

In this review article, all the anticancer activities reported so far for garcinol are compiled to highlight the promising therapeutic potential of this fascinating natural agent present its pleiotropically regulation of cellular processes in cancer. In addition to analyzing its chemopreventive and chemotherapeutic properties, the possibilities to improve the bioavailability of garcinol in the human body by applying nanotechnological approaches are also under consideration in this review to further promote clinical trials with this potent phytochemical.

## 2. Chemistry of Garcinol

**Figure 1:**
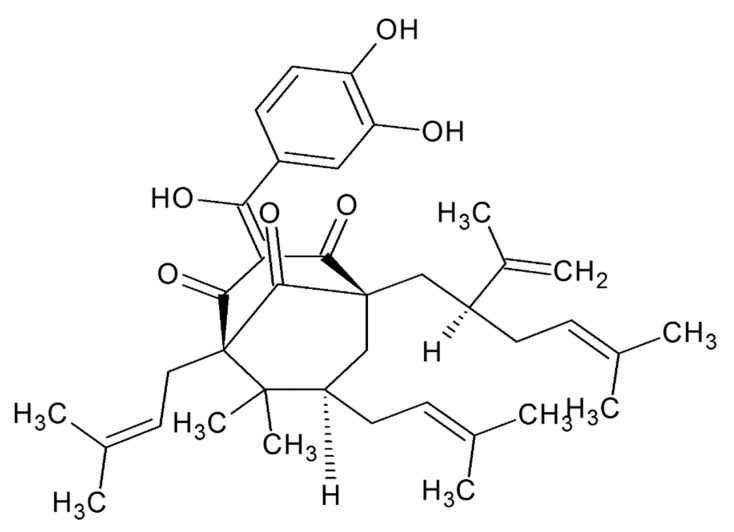
Chemical structure of garcinol.

Chemically, garcinol is polyisoprenylated benzophenone (Figure 1) mainly found in *Garcinia indica* (Kokum) and *Garcinia cambogia* (Malabar tamarind) of the Mangosteen and Clusiaceae families, respectively [22]. Methanol extraction of dried *G. indica* plums resulted in the promising extraction of garcinol [9]. Further, re-dissolution of the above prepared extract in ethyl acetate followed column chromatography on silica gel. From hexane elute, yellow needle-like crystals of garcinol with a melting point of 132 °C have been reported [9,23]. Another method comprises the initial removal of hydroxycitric acid from plant fruits. Then, methanol extract was prepared and adsorbed on Celite and extracted with hexane. Finally, the hexane extract was purified by column chromatography [9].

Moreover, garcinol has also been reported by chemists from *G. multiflora*, with an yield of 0.5 gm per 5.2 kg dried fruits [24,25]. Other *Garcinia* species, including *G. morella*, *G. yunnanensis*, *G. xanthochymus*, *G.travancorica,* and *G. buchananii*, have also been considered a promising source of garcinol [26,27,28,29,30].

### Structure Activity Relationship

The chemical structure of garcinol was first described by Sahu and his colleagues in 1989 [31]. Garcinol’s structure is similar to that of curcumin (a well-presented natural anti-inflammatory, antioxidant), tumor inhibitor hydroxybenzoyl, and a enolic β-diketone group [31,32].

The C-3 ketonic group and the phenolic ring group with hydroxyl are the primary oxidation sites of garcinol, which are also biologically active in its oxidized products during metabolism transformation [33].

The 1,2 carbon double bond of α,β unsaturated ketone has also been found to be critical for apoptosis-inducing activity and for garcinol’s cytotoxicity [33]. A principal site of the antioxidation of garcinol is the double bond in the isoprenyl ring. Nonetheless, compounds without this substitution have been found to have active antioxidants that have structural resemblances to garcinol, such as curcumin [34]. The garcinol isoprenyl chain has hydrophobic faces, which are important to biological targets [33].

The C8 side chain is one of garcinol’s main functional groups with anti-cancer effect [35]. The bioisosteric substitution of fluoro groups in garcinol instead of phenolic hydroxyls can increase their bioavailability and improve their metabolic rates [36].

## 3. Anti-Neoplastic Regulation of Cellular Processes in Tumor

### 3.1. Apoptosis and Cell Cycle Arrest

**Figure 2:**
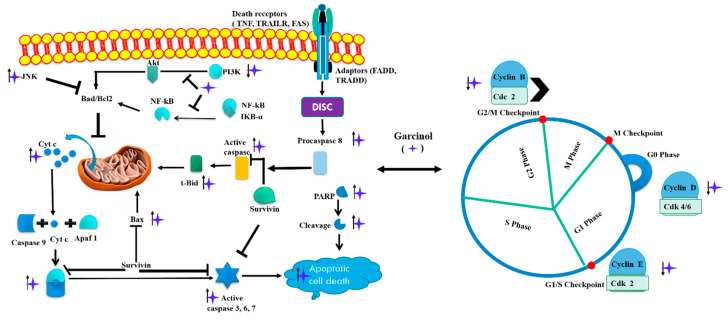
Schematic representation of apoptosis with garcinol treatment (↑ up regulation, ↓ down regulation). Garcinol leads to cell cycle arrest in G0, G1/S, and G2/M phase via regulating binding of CDKs and cyclin molecules (Cyclin B, D and E) respectively. Garcinol leads to apoptosis via PI3K/AKT and NF-κB signaling pathways, PARP cleavage and activation of caspases. Phosphoinositide 3-kinase (PI3K), AKT serine/threonine kinase (Akt), cyclin-dependent kinases (CDKs), poly(ADP-ribose) polymerase (PARP), nuclear factor kappa B (NF-κB), Bcl-2 associated X, apoptosis regulator (Bax), inhibitor of kappa B (IκB-α), Cytochrome c (Cyt c), Cyclin dependent kinase 1 (Cdc2), ppoptotic protease activating factor 1 (Apaf-1), truncated BH3 interacting domain death agonist (tBid), Bcl-2 associated agonist of cell death (Bad), B-cell lymphoma 2 (Bcl-2), c-Jun N-terminal kinase (JNK).

Induction of apoptosis and cell cycle arrest are considered to be very important molecular mechanisms governed by phytochemicals (Figure 2) [37]. For instance, a report on medulloblastoma, a platelet-derived growth factor receptors (PDGFRs) signaling pathway, was implicated in tumor progression. The authors have shown that garcinol treatment in mouse embryo fibroblast cells was able to arrest the cell cycle in S phase and cause apoptosis, mediated by caspase activation and suppression of cyclin A and E. Mechanistic insight provided by the authors elaborate on the potential of inhibiting PDGFR signaling by garcinol in medulloblastoma in vitro [38]. Furthermore garcinol treatment also suppressed oral squamous cell carcinoma (OSCC) cells proliferation, cell cycle, migration and invasion, and colony formation [39]. In another report involving the treatment of garcinol on SCC-4, SCC-9, and SCC-25 OSCC cells for 48 h indicated the role of cyclooxygenase-2 (COX-2) and NF-κB in cell growth and progression. The authors showed that treatment with garcinol significantly induced apoptosis and cell cycle arrest probably mediated by downregulation of COX-2 and NF-κB [40]. Comparative analysis between the apoptosis inducing effects among garcinol and 8-allyl garcinol in CAL27 OSCC cells suggest garcinol to be more potent. Moreover apoptotic effect of garcinol was significantly higher at both 10 μmol/L and 20 μmol/L concentrations in comparison to 8-allyl garcinol [41]. Molecular mechanism on the effect of garcinol in cervical cancer points towards the role of T-cadherin. The authors suggest that downregulation of T cadherin is linked with tumorigenesis. It was reported that treatment with garcinol in cervical cancer cell line (HeLa and SiHa) at different concentration points led to an increase in T-cadherin levels. This increase was attributed to inhibition of cell cycle inhibition and apoptosis through activation of T-cadherin/P13 K/AKT signaling [42].

The majority of investigations on the anti-neoplastic effect of garcinol have been conducted in breast cancer, as summarized in Table 1 and Table 2. Garcinol has been reported to initiate apoptosis in MCF7, MDAMB231, and SKBR3A breast cancer cell lines via down regulation of B-cell lymphoma-extra-large (Bcl-XL) and p53-dependent Bcl-2 associated X apoptosis regulator (Bax) induction [43]. Garcinol is a known p300/CBP associated factor (PCAF) inhibitor, which has also been shown to destabilize alteration/deficiency in Activation 3 (ADA3) in a proteasome dependent manner. ADA3 is involved in cell cycle progression and stability. Destabilization of ADA3 by garcinol treatment in HER2+ breast cancer cells leads to cell cycle inhibition and apoptosis via receptor tyrosine kinase (RTK)-AKT-p300-ADA3 signaling pathway and supported with accumulation of p27, reduction in pH(S10), proliferation cell nuclear antigen (PCNA), and induction of cleaved poly(ADP-ribose) polymerase (PARP) [44]. Another report suggests the loss of mitochondrial fragmentation and its transmembrane potential (ΔΨm) and in support of garcinol’s pro-apoptotic effects conducted in MCF-7 cells. Moreover, the authors have suggested the induction of NADPH oxidase 1 (NOX1) and the dissociation of apoptosis signal regulated kinase 1 (ASK1) and Thioredoxin 1 (Trx1) in conjunction as the possible mechanism involved [45]. Similar to this report, another investigation reports the induction of caspase-independent mitochondrial pathway supported with experimental evidence showing increase in Bax/B-cell lymphoma 2 (Bcl-2) ratio and translocation of apoptosis inducing factor.

Furthermore, the authors suggested the induction of the ASK1-mitogen activated protein kinase 4/7 (MKK4/MKK7)-c-Jun N-terminal kinase (JNK)/stress activated protein kinase (SAPK) signaling pathway by garcinol treatment in MCF-7 cells and xenograft models as the possible mechanism involved [46]. Estradiol treatment inhibits apoptosis and promotes cell proliferation in MCF-7 breast cancer cells. However, it has been shown that garcinol treatment inhibited estradiol induced cell proliferation and induced apoptosis via cell cycle arrest in G0/G1 phase. Probable mechanism included the decreased levels of acetylated p65 in NF-κB pathway leading to downregulation of cyclin D1, Bcl-2, and Bcl-xl [47]. Probable involvement of NF-κB pathway in garcinol mediated anticancer effects has also been reported in triple negative breast cancer cell lines and xenograft models. The authors reported that garcinol treatment leads to deregulated Wnt and NF-κB signaling cascade and upregulation of miR-200s, let-7s microRNA family. Moreover the upregulation of miRNA’s turnaround of epithelial-to-mesenchymal (EMT) transition induced by garcinol in BT-549 and MDA-MB-231 breast cancer cells [48,49]. In lung cancer, garcinol induces apoptosis by impairing phosphorylation of low-density lipoprotein receptor-related protein 6 (LRP6) and down regulating expression of Axin2, β-catenin, cyclin D1, and disheveled segment polarity protein 2 (Dvl2) in H441 and A549 NSCLC cell lines.

Moreover, garcinol significantly reduced sphere and colony formation. The authors suggested garcinol-mediated inactivation of STAT3 and regulation of Wnt/β-catenin signaling as the probable mechanism involved in modulation of the liver cancer stem cell (LCSC) phenotype [50]. p53 levels may modulate and direct the garcinol-induced anti-cancer mechanistic. In other report, it was shown that garcinol in a dose dependent manner significantly arrested the H1299 (p53-null) cells in G1 stage whereas it induced apoptosis in H460 (p53-wild type) cells. Furthermore, garcinol treatment also led to differential levels of cyclin dependent kinases (CDK). Cyclin D1, cyclin D3, CDK2, and CDK4 levels were downregulated, whereas CDK6 and cyclin E were upregulated in H1299 cells. Overall, the authors suggested that p53-independent effects of garcinol may be mediated through p38-mitogen activated kinase like protein (MAPK) signaling and upregulation of p21 (Waf1/Cip1) [51]. In colorectal cancer, garcinol has been reported to act through microsomal prostaglandin E synthase-1 (mPGES-1)/prostaglandin E synthase 2 (PGE2)/hypoxia inducible factor-1 alpha (HIF-1α) axis in HT-29 cells. Garcinol was reported to cause apoptosis probably mediated through caspase 3 activity and downregulation of HIF-1α, C-X-C chemokine receptor type 4 (CXCR4), vascular endothelial growth factor (VEGF), matrix metalloproteinase-2 (MMP-2), and matrix metalloproteinase-9 (MMP-9) [52].

Similar, garcinol has reported to induce apoptosis in via caspase 3 activity in human HCT-116 and HT-29 colon cancer cells at 2–10 µM. However the authors suggest that these effects by garcinol are only observed at high concentration and treatment at low concentration (<1 µM) may have growth stimulating effects [53]. Another possible pathway to support the apoptotic potential of garcinol was reported in HT-29 cells. It was shown that garcinol (20 µM) modulates the ratio of Bcl-2 and Bax, which was found to be associated with cytochrome c (cyt c) release and PARP cleavage [54]. Utilizing flow cytometry based analysis, garcinol was reported to induce apoptosis and arrest human HL-60 promyelocytic leukemia cells in G2/S phase accompanied with disruption of mitochondrial membrane potential in a concentration dependent end points [55]. Involvement of caspase 3 and disruption of mitochondrial membrane potential in anti-proliferative and apoptotic inducing potential of garcinol has also been reported in four other leukemia cell lines [56]. Additionally, garcinol (20µM)-induced apoptosis in HL-60 cells via degradation of PARP and accompanied with release of procaspase-9 and cytochrome c along with of caspase-2, caspase-3, Bax, and Bcl-2 associated agonist of cell death (Bad) [57].

Garcinol activates p-PI3K/AKT and phosphorylated mechanistic target of rapamycin kinase (p-mTOR) pathway, thus priming PC-3 cells directly toward apoptosis. Further supportive study in xenograft mouse model showed 80 percent reduction of tumor size [58]. In an another report, downregulation of NF-κB signaling pathway was implicated in garcinol induced apoptosis in LNCaP, C4-2B, and PC3 prostate cancer cells [59]. STAT family of genes, particularly the STAT3 pathway, is also modulated by garcinol for its apoptotic activities and, the observation has also been correlated in xenografted tumor of hepatocellular carcinoma (HCC). In silico analysis suggests the probable ability of garcinol to bind with Src homology 2 (SH2) domain of STAT3 and block its dimerization [60]. Involvement of p53 in apoptotic pathway remains an interesting observation point. In an another report including Hep3B (p53 lacking) cells, garcinol treatment caused apoptosis and correlated with increased levels of Bax/Bcl-2 ratio, caspase-3,caspase-8,caspase-9, PARP cleavage, and reduced mitochondrial membrane potential in a time- and dose-dependent end points [61].

### 3.2. Antioxidant and Anti-Inflammatory

**Figure 3:**
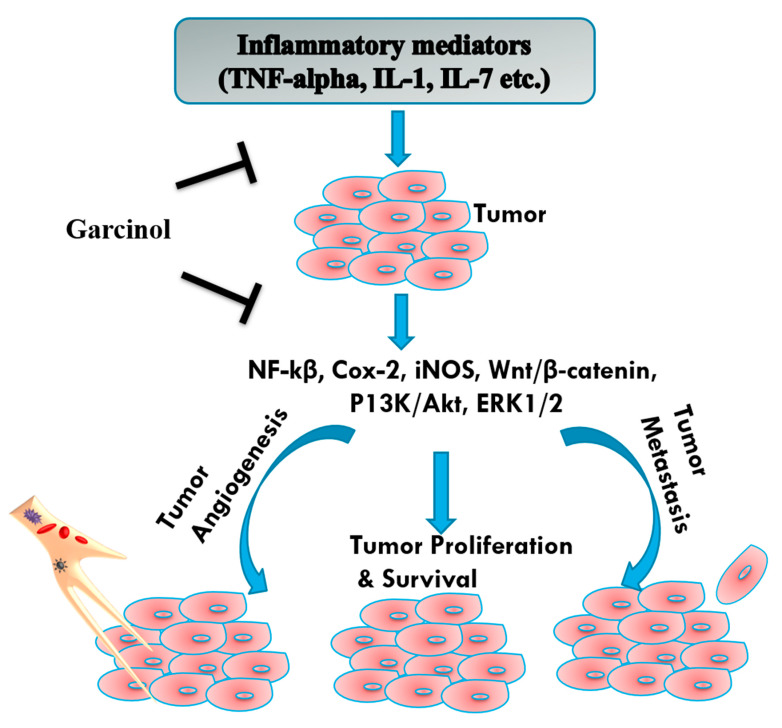
Garcinol mediates anti-inflammatory role in tumor cells via TNF-α, IL-1, and IL-7 which in turn modulates anti-angiogenic and anti-metastatic action in tumor cells is mediated via NF-κB, Cox-2, iNOS, PI3K/AKT, ERK1/2, and Wnt/β-catenin signaling pathways. Tumor necrosis factor-alpha (TNF- α), Interleukin-1 (IL-1), Interleukin-7 (IL-7), Cyclooxygenase-2 (COX-2), inducible nitric oxide synthase (iNOS), nuclear factor kappa B (NF-κB), phosphoinositide 3-kinase (PI3K), AKT serine/threonine kinase (Akt), extracellular signal regulated kinases 1/2 (ERK1/2), wingless (Wnt).

Unregulated expression of inflammatory mediators such as cytokines and chemokines are considered a major cause of inflammation [43,62,63,64,65,66]. As a result of inflammation, excess of free radicals like reactive nitrogen species (RNS) and reactive oxygen species (ROS) are produced [67,68]. These free radicals lead to the induction of oxidative stress in body leading to the progression of various diseases like cancer, inflammatory diseases, cardiovascular diseases, and atherosclerosis [14,40,69,70,71]. The cancer development and efficiency of chemotherapy is affected by the inflammation caused in the body [72,73]. Therefore, it is of great consideration to study the anti-cancer agents and their cellular mechanism of action in blocking the cancer cells’ proliferation and inflammation inhibition [74,75,76,77,78]. In comparison to the synthetic drug formulations, plants are known to cause minimum side effects and are exhibit enhanced antioxidant and anti-inflammatory properties [43]. One such example of plant-derived formulation possessing anti-cancerous, anti-inflammatory and antioxidant properties [11,33,79] is garcinol, isolated from the rind of fruits of *Garcinia indica*. Garcinol has proved anti-cancerous properties against various types of cancers like colon, prostate, leukemia, breast, and pancreatic cancer [31]. These anti-cancerous properties are attributed due to the anti-inflammatory and antioxidant properties of garcinol (Figure 3) [11,43].

**Figure 4:**
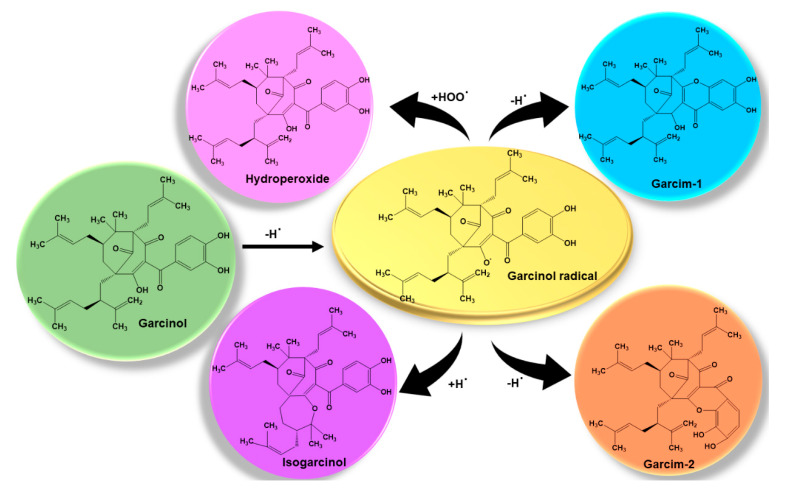
Schematic representations of garcinol and its derivatives in response to free radicals.

The antioxidant properties of garcinol may be attributed to its β-diketone and catechol fragments in its structure [80]. In response to garcinol the free radicals get converted (Figure 4) to derivatives like garcim-2, isogarcinol, xanthone derivative gatcim-1, and hydroperoxide metabolite to neutralize oxidative stress caused by them [53,81,82]. The tumor cells are suppressed by garcinol by blocking the activity of NF-κB [10]. Studies have shown that garcinol suppresses the colon cancer by inhibiting COX-2 and inducible nitric oxide synthase (iNOS) activities [83]. In addition, the inhibition of COX-2 and NF-κB activities by garcinol also attributes to its anti-inflammatory properties [84,85]. Garcinol is known to possess antioxidant properties greater than (+)-catechin in scavenging superoxide anion (O^2−^) [86] and dl-α-tocopherol in scavenging 1,1-diphenyl-2-picrylhydrazyl (DPPH) [87]. Garcinol helps in preventing the DNA damage caused by OH^.-^ by quenching OH^.-^ at IC_50_ of 0.32 μM [86] and can also quench 100 μM peroxynitrite [88]. The introduction of garcinol in proliferating cancer cells leads to apoptosis via release of cyt c from mitochondria and activation of caspase-2/3/9 [57]. Caspase-3 activation with the introduction of garcinol was observed in intestinal tumor cells [53] and breast tumor cells [49].

The anti-inflammatory effect of garcinol could also be illustrated through its interference with various inflammatory cascades. One of its pathways for anti-inflammatory effect is mediated via NF-κB activation [89]. The second pathway involves the interference of garcinol with lipopolysaccharide (LPS)-mediated phosphorylation of ERK1/2 and reduction of COX-2 products and thus the reduction of prostaglandins [90]. These anti-inflammatory and antioxidant properties of garcinol make it an important anti-cancer agent. The anti-cancer effects are produced through cell cycle arrest stimulation, apoptosis, cancer cell metastasis prevention, and inhibition of cell proliferation and growth [91,92]. In a study, garcinol exhibited the reduced tumorigenic cell proliferation in human pancreatic BxPC-3 cell lines (IC_50_ value of 20 μM) and human lung carcinoma cell lines (IC_50_ value of 10 μM) by inhibiting downstream synthesis of prostanoid and COX-2 expression [91,92].

In another study, Wnt/β-catenin P13K/Akt and ERK1/2 signaling were inhibited via down regulation of PCNA, COX-2, and iNOS by garcinol in colon carcinogenesis-associated inflammation in mice [93]. Isogarcinol (isoform of garcinol) is also known to possess the anti-inflammatory properties as it suppresses collagen-induced arthritis in vivo and down regulates NF-κB, iNOS, and COX-2 [94]. The studies listed here, as well as various other studies, have indicated that garcinol is a valuable plant-based component possessing anti-inflammatory and antioxidant properties that make it a suitable anti-cancerous agent (Table 1 and Table 2).

### 3.3. Angiogenesis and Metastasis

Garcinol, a histone acetyltransferase (HAT) inhibitor, is extensively studied to induce apoptosis and cell cycle arrest in cancer cells. Besides, apoptotic activity garcinol has also been reported to modulate both angiogenesis, as well as metastasis, which are key hallmarks of a cancer cell [95,96]. The present scientific evidence supporting (Table 1 & Table 2) anti-angiogenic and anti-metastatic potential of garcinol in cancer cells are summarized hereafter.

**Figure 5:**
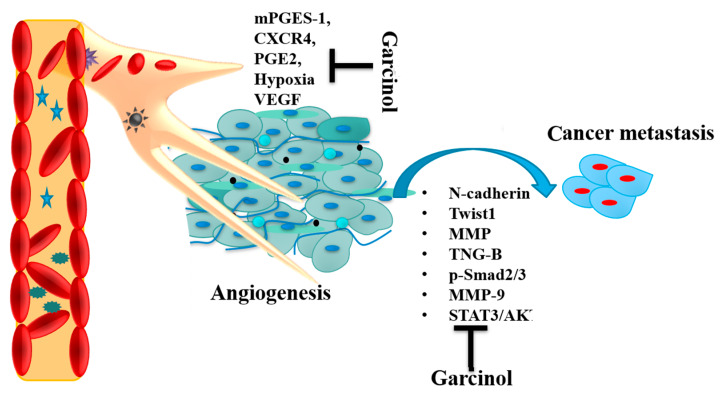
Anti-angiogenic and anti-metastatic action of garcinol. Microsomal prostaglandin E synthase-1 (mPGES-1), prostaglandin E synthase 2 (PGE2), hypoxia inducible factor-1 alpha (HIF-1α), C-X-C chemokine receptor type 4 (CXCR4), vascular endothelial growth factor (VEGF), matrix metalloproteinases (MMP-9), AKT serine/threonine kinase (Akt), signal transducer and activator of transcription 3 (STAT3), twist-related protein 1 (Twist1).

Garcinol, a polyisoprenylated benzophenone has been seen to modulate multiple pro-inflammatory signaling pathways leading to suppression of angiogenesis in malignant cells (Figure 5) [97,98,99,100,101,102]. In one of the studies carried out by Li et al. (2013), garcinol was reported to deregulate STAT3/NF-κB activation, an oncogenic transcription factor in head and neck carcinoma (HNSCC) in a dose and time-dependent manner. This inactivation of STAT3/NF-κB was coupled to suppression of upstream kinases i.e., janus kinase 1/2 (JAK1/2), c-Src, TGF-β-activated kinase 1 (TAK1), and inhibitor of IκB kinase (IKK) in HNSCC cells. Upon intraperitoneal administration in HNSCC xenograft mice model, garcinol was reported to inhibit growth in an athymic nu/nu mice [103]. Similar findings were reported by another group in SCC-4, SCC-9, and SCC-25 human OSCC cells, wherein garcinol was shown to inhibit angiogenesis and tumor proliferation via inhibition of NF-κB pathway (decreased NF-κB and COX-2 expression) [40]. In yet another study, carried out in HCC garcinol was shown to inhibit inteleukin-6 (IL-6)-inducible STAT3 dimerization and acetylation by binding to SH2 domain of STAT3, which in turn suppressed the growth of HCC [60]. In human colon cancer cells (HT-29), garcinol treatment was shown to inhibit angiogenesis and proliferation via downregulating expression of mPGES-1, HIF-1α, CXCR4, MMP-2, VEGF, and MMP-9 via suppression of mPGES-1/PGE2/HIF-1α signaling pathway [52]. In triple negative breast cancer cells, garcinol was reported to sensitizes tumor cells to Taxol, which was mediated via suppression of caspase-3/cytosolic CA^2+^-independent phospholipase A2 (iPLA_2_) and NF-κB/Twist-related protein 1 (Twist1) pathway in mouse 4T1 breast tumor model [21].

In one of the initial reports on the anti-metastatic potential of garcinol in pancreatic adenocarcinoma cells, garcinol was seen to inhibit metastasis via decreasing expression of MMP-9, VEGF, PGE2, and inteleukin-6 (IL-8) in PaCa human pancreatic cancer cell lines [92]. Another study by Duan and colleagues (2018) tested the anti-proliferative and anti-invasive effect of garcinol in GBC-SD and NOZ human gallbladder carcinoma cells (GBC). Garcinol treatment was reported to impede growth and invasion of GBC cells in a dose- and time-dependent manner via downregulation of MMP-2/9 expression levels, which lead to suppression of STAT3/AKT pathway in GBC SD cells [104]. In a recent study by Wang et al. (2020), garcinol was shown to inhibit metastasis in esophageal cancer both in vitro and in vivo via downregulation of transforming growth factor beta 1 (TGF-β1) and p300 signaling cascade (Table 1 and Table 2) [105]. The study reported that garcinol inhibited migration and invasion in KYSE150 and KYSE450 human esophageal tumor cells in a dose-dependent manner (5–15 μM). This dose-dependent inhibition lead to suppression of TGF-β1 and decrease in p-Smad2/3 nuclear expression along with p300/CREB binding protein (CBP) protein levels which impeded tumor metastasis. In a pulmonary metastasis in vivo mouse model, garcinol (20 mg/kg) administered intraperitoneally, or 5-FU (20mg/kg) led to significant decrease in lung tumor nodules and Ki-67, p300, and p-Smad 2/3 expression in lung tissues.

### 3.4. MicroRNAs (miRNA)

In addition to the cellular mechanism regulated by garcinol, it has also been reported to mediate its regulatory effect via expression of miRNAs. In the last decade, the role of miRNAs has emerged significantly wherein the alterations in miRNA expression has been shown to regulate anticancer effects in tumorigenic cells. In context of garcinol, we have limited studies, which have explored the plausible mechanism of action of garcinol with respect to miRNAs. The available literature is being summarized in this section. In an interesting study by Farhan and colleagues [20], garcinol was reported to reverse EMT in A549M and H1299 cells via mechanistic action of miRNAs. Garcinol was reported to upregulate let-7c, miR-200b, miR-205, and miR-218, the key miRNA molecules which reversed EMT [20]. In MDA-MB-231 and BT-549 breast cancer cells, garcinol was reported to regulate EMT via deregulation of let-7 and miR-200 coupled to NF-κB and Wnt signaling pathway [48]. In PANC-1 human pancreatic cancer stem-like cells, garcinol was reported to inhibit the metastatic potential of PANC-1 stem cells via downregulating Bcl2 family member (Mcl-1), enhancer of zeste 2 polycomb repressive complex 2 subunit (EZH2) and GLI family zinc finger 1 (Gli-1) which was coupled to upregulation of miR-200c and downregulation of Notch1 signaling [112]. In yet another study conducted on PaCa pancreatic cancer cells, garcinol was reported to synergize with gemcitabine and the cumulative effect induced apoptosis and inhibited cell proliferation via modulation of miR-21, miR-495, miR-638, miR-453, miR-196a, and miR-605 [113]. This synergistic effect induced apoptosis and inhibited PaCa cell proliferation via modulation of NF-κB, VEGF, PARP, MMPs, interleukins, and caspases expression [113]. In view of limited studies on associated mechanism of action via garcinol specific miRNAs, it will be too early to comment on the garcinol-miRNA effects. However, in view of promising findings from the initial studies, this does lead the way ahead for further research into looking at miRNAs activated or downregulated through garcinol treatment and their effect on cancer cells.

## 4. Synergistic Effects of Garcinol

The standard chemo-preventive measures used for cancer treatment involves marginal survival advantage with multiple side effects and drug resistance [116]. Alternative effective therapeutic strategies include the use of naturally occurring bioactive components that have been exploited for medicinal purposes, in combination with established chemotherapeutic drugs [113]. Combinatorial or synergistic therapeutic strategies, thus, have been manifested not only to reduce the levels of required chemotherapy dosage but also potentiates the anticancer effect of standard drugs [116]. Garcinol, has been well distinguished to possess potential anti-cancer properties attributed to the ability to inhibit HATs, NF-κB and STAT [33]. Furthermore, this pleotropic compound’s activity spectra includes antibiotic [117], anti-inflammatory [118], antioxidant, antidepressant, and anxiolytic [119] properties via modulation of key regulatory cell signaling cascades.

This bioactive compound garcinol has been documented to exhibit potent synergism with curcumin and thus inhibit the growth of human BxPC-3 and Panc-1 pancreatic cancer (PaCa) cells in a dose-dependent manner [120]. In addition, garcinol has also been found to enhance the chemo preventive potentiality of various other therapeutic drugs. The compound has been known to synergize with a standard cytotoxic chemotherapeutic agent, gemcitabine to inhibit cell proliferation and influence apoptosis in PaCa cells with significant manipulation of important cancer regulators including PARP, VEGF, MMPs, interleukin’s caspases, and NF-κB [113]. Further, miRNA biomarkers specific to chemo-protective effects of garcinol, which sensitize PaCa cells to gemcitabine treatment were also identified [113].

Recently, it was noted that combinatorial treatment of garcinol with tumor necrosis factor related apoptosis inducing ligand (TRAIL) significantly induced apoptosis in SK-Hep1 hepatoma cells, A549 lung carcinoma cells and A498, Caki and ACHN renal carcinoma cells by triggering down-regulation of c-FLICE-like inhibitory protein (c-FLIP) and up-regulation of DR5 at post-translational levels and that too without altering cell viability in normal cells [121]. Garcinol had also been reported to inhibit the viability of various HNSCC cell lines, and enhance cisplatin-induced apoptosis in a human xenograft HNSCC mouse model through down-regulation of NF-ĸB expression [10].

## 5. Bioavailability and Nanotechnology Studies of Garcinol

Garcinol has extremely low bioavailability and fast metabolic clearance [31]. Very limited studies on the bioavailability of garcinol have been performed. According to one study, effect of garcinol was more efficient in in vitro cell culture system with lack of serum. Further, the addition of 10% fetal serum decreased half maximum inhibitory concentration value by 10 times and can also use for HCT116 growth [19]. Similar effects have been shown to reflect the association between garcinol’s interaction with serum proteins [19]. The new method for treating cancer and other inflammatory disorders has been gradually attracted in recent years towards the nanotechnology [122,123]. The choice of an ideal encapsulation polymer is important because it controls key properties, such as solubility, capacity for loading drugs, stability, and drug release profile [124,125].

In vitro and in vivo experiments using garcinol showed lesser bioavailability, poor aqueous solubility and low therapeutic performance, which encouraged researchers to develop a new nanoparticle garcinol system in order to enhance its aqueous solubility, bioavailability, and potential therapeutic effectiveness [11]. Garcinol was investigated by differential calorimetry (DSC), Fourier transform infrared, and ray diffraction in nanoparticles. The nanoparticles have been characterized by morphology, particle size, zeta potential, encapsulation efficiency, polydispersity index, and in vitro release profile [124]. However, World Health Organization (WHO) studies also highlighted the need for emergency pharmaceutical intervention to develop new and creative methods to control multidrug resistance bacterial strains [126]. A potential candidate for the prevention of bacterial infections has recently been focused on antimicrobial nanoparticles [22].

Garcinol has shown a wide spectrum of bioactivities including antimicrobial, antioxidants, anticancer, and anti-inflammatory activity. Nanoparticles of titanium dioxide are commonly used for medical use and are known to be of important photocatalytic activity [127]. In order to enhance antibacterial activity, garcinol has been used to enhance the surface of titanium dioxide nanoparticles. Garcinol-coated poly(lactic-co-glycolic acid) (PLGA) nanoparticles consist of poly(lactic-co-glycolic acid) biodegradable and a bio-compatible, successfully developed polymer. PLGA nanoparticles are internalized by pinocytosis and endocytosis in cells [128]. d-α-tocopheryl polyethylene glycol 1000 succinate (TPGS)-emulsified PLGA nanoparticles loaded by garcinol vitamin E have been prepared by nanoprecipitation [124]. The conclusion was that garcinol-loaded nanoparticles provide maximum solubility and improve the bioavailability of garcinol.

Silver nanoparticles (AgNPs) receive significant attention among many forms of metal nanoparticles because of their high antimicrobial properties. They bind and anchor to the cells’ surface and interfere in a number of cells, leading to cell apoptosis [129]. A well diffusion test, broth microdilution assay, and time kinetic analysis were used to determine the antimicrobial activity of garcinol silver nanoparticles. The synthesized nanoparticles have antimicrobial activity that depends upon several factors, such as their size and shape, and the various modes of action induced by the existence of garcinol and silver nanoparticles [22]. The median size of the nanoparticles enhances the biocompatibility and stability [22]. Such particles also have a high surface to volume ratio because of their smaller size, which allows for more efficient antimicrobial activity and interaction with micro-organisms. Fernando et al. conclude that the preparation of nanoparticles has improved the solubility of garcinol in water where silver nanoparticles enable garcinol to be tested to detect their mutual therapeutic effectiveness [22].

## 6. Conclusions and Future Perspective

Garcinol, a potent antioxidant and anti-inflammatory compound, mediates its anti-cancer effects via modulation of different cellular processes, i.e., anti-inflammation, apoptosis induction, cell cycle arrest, and inhibition of angiogenesis and metastasis via modulation of signaling cascades and gene expression. Although promising results have reciprocated from pre-clinical studies, the development of garcinol as an anti-cancer agent in therapeutics is mainly limited by the lack of understanding of systematic pharmacokinetic evaluation of garcinol. This is essential to determine and estimate the effects of the appropriate dosage on its pharmacodynamics characteristics. This requires the precise establishment of the appropriate dose, the route of administration, tolerability, potency, absorption, and bioavailability under physiological conditions along with the characterization of a therapeutic index, which has yet not been addressed. Additionally, the toxicology profile of garcinol still remains elusive. Although garcinol extract has been extensively used for centuries and is considered safe, a major challenge is to establish the systematic toxicological study of purified form of garcinol. This necessitates the need to understand the pharmacological profile of garcinol. This enhanced understanding of garcinol from an empirical platform to evidence-based will translate into clinically applicable pharmacotherapy. Hence, the present ongoing research in the dimension of exploring the precise metabolic mechanism of action of garcinol will help to address the unknown dimensions in the practical translation of garcinol into clinical studies.

## Figures and Tables

**Table 1:** Anticancer effects of garcinol based on in vitro studies.

Type of Cancer	Cell Lines	Effects	Mechanisms	Concentration	Ref.
Skin (Melanoma)	B16F10 cells	Inhibited cell proliferation	↑ Apoptosis, ↑ Caspase-3, ↑ Caspase-9, ↑ Bax, ↓ Bcl-2,	10–50 μg/mL	[85]
Head	CAL27	Reduction of cell viability	↑ Apoptosis, ↓ STAT3, ↓ c-Src, JAK1/2, ↓ NF-κB, ↓ TAK1	0–50 µM	[103]
Brain (Glioblastoma)	U-87 MG and GBM8401 GBM	Inhibited cell viability	↑ Apoptosis, ↑ STAT3 and ↑ STAT5A, ↑ hsa-miR-181d/STAT3 and hsa-miR-181d/5A ratio, ↑ Bax; ↓ Bcl-2	2.5–40 µM	[106]
Cervical	Hela and SiHa	Suppressed cell viability, invasion, and migration	↑ Apoptosis ↑ T-cadherin knockdown of T-cadherin, ↑ P13K/AKT signaling pathway	0, 5, 10, 25 μM	[42]
Oral squamous cell carcinoma	SCC-4, SCC-9 and SCC-25	Inhibits tumor cell proliferation, cell cycle progression, and angiogenesis	↓ NF-κB ↓ COX-2 ↓ VEGF	0 to 25 µM	[40]
	Cal 27	Inhibit cell proliferation	↑ Apoptosis	0–20 µM	[41]
Esophageal	KYSE150 and KYSE450	Inhibits cancer metastasis	↓ p300 and TGF-β1, ↓ p300/CBP, ↓ p-Smad2/3	0, 5, 10, 20 μM	[105]
Breast	MCF-7, MDA-MB-231, AU-565	Inhibition of cell proliferation	↓ cyclin D3 expression ↓ a9-nAChR expression	0 to 20 μM	[107]
	MCF7	Suppressed cell proliferation	Inhibit histone acetyltransferase activities, ↓ acetylation of p53, ↑ DNA damage signaling and the induction of chromatin regulators such as TIP60 and SUV420H2	0, 10, 20 μM	[108]
	MDA-MB-231 and BT-549	Inhibit cell proliferation	↑ E-cadherin, ↓ vimentin, ↓ ZEB-1, ↓ ZEB-2, ↑ miR-200, ↑ let-7 family microRNAs	0 to 25 µM	[48]
	MDA-MB-231	Inhibit cell proliferation	↑ Apoptosis, ↑ STAT3, ↓ total and p-STAT-3, ↓ IL-6-induced STAT-3 phosphorylation, ↓ u-Pa, ↓ VEGF and MMP-9	0, 10, 25 µM	[109]
	MCF-7	Inhibited cell proliferation, inhibited cell cycle progression	↑ Apoptosis, Cell cycle arrest at G0/G1 phase, ↑ ac-H4, ↑ ac-H3, ↑ NF-κB/ac-p65, ↓ ac-p65 in NF-κB pathway, ↓ cyclin D1, ↓ Bcl-xl, ↓ Bcl-2	10–50 µM	[47]
	MCF7, MDAMB231 and SKBR3	Anti- proliferative responses	↑ Apoptosis, ↑ P53 ↑ Bax ↓ Bcl-XL, ↓ Nitrite and TNF-α level	1.56–25 μg/mL	[43]
Leukemia	HL-60 cells	Cancer chemo preventive effect	↑ Apoptosis, ↑ caspase-3/CPP32 activity, ↑ degradation of PARP, ↓ Bcl-2, ↑ Bad, ↑ Bax	IC50 values of 9.42 µM and 19.5 µM	[57]
	NB4, HL60, U937, and K562	Growth inhibitory effects	↑ Apoptosis, ↑caspase 3, ↓ mitochondrial transmembrane potential	0–20 µM	[56]
Lung	H441 and A549 NSCLC cell lines	Inhibits cancer cell	↓ Wnt/β-catenin /STAT3, Impaired phosphorylation of LRP6, ↓Axin2, ↓ β-catenin, Dvl2, ↓ cyclin D1,	0~20 μM	[50]
	A549 and H1299	Anti- proliferative responses	↑ Apoptosis, ↑ miRNAs (miR-200b, miR-205, miR-218, and let-7c)	0–20 µM	[20]
	A549, H460, H1299, H1650, H358, and HCC827	Inhibited cellproliferation/suppressed cellviability	↓ C/EBPβ, ↓ ALDH1A1, ↑ DDIT3	0.1% (*v*/*v*)	[110]
Hepatocellular	A431, Hep3B cells	Decreasescell viability	↑ Apoptosis, ↓ CEBP, ↑ GADD153, ↑ ROS, ↓ mitochondrial membrane potential, ↑ Bax/Bcl-2 ratio, ↑ Caspase-8, ↑ tBid, ↑ caspase-3, ↑caspase-9	0–50 µM	[61]
	MH1C1 and HepG2	Inhibition of cell proliferation	↑ Apoptosis, ↓ cyclin E, ↓ cyclin B, ↓ bcl-2	500 ppm	[111]
	C3A, HepG2, PLC/PRF5, and HUH-7	Inhibition of cell proliferation	↑ Apoptosis, ↓ IL-6, ↓ STAT3 acetylation, ↓ cyclin D1, ↓ Bcl-2, ↓ Bcl-xL, ↓ survivin, ↓ Mcl-1, ↓ VEGF	10 µM	[60]
Gastric	RAW264.7 macrophage cell lines	Chemoprevention and anti-proliferative responses	↓ (iNOS) ↓ COX-2, lowers the LPS-induced increase of intracellular ROS	0–5 µM	[89]
Pancreatic	BxPC-3	Inhibit cell proliferation	↑ Apoptosis, ↓ STAT-3 signaling pathway, ↓ IL-6, ↓ uPA, ↓ VEGF, ↓ MMP-9	0, 10, 25 µM	[109]
	BxPC-3	Inhibited cell growth	↓ NF- κB signaling pathway	0–25 µM	[59]
	PANC-1	Suppresses oncogenic properties of cells	↑ ABCG2, ↑ Oct4, ↑ CD44, ↓ Mcl-1, ↓ EZH2, ↓ Gli-1, ↓ Notch1, ↑ tumor suppressor miRNAs, ↑ miR-200c	0–10 µM	[112]
	BxPC-3 and Panc-1	Inhibited cell proliferation	↑ Apoptosis, ↑ PARP, VEGF, MMPs, ILs, caspases, and NF-B, ↓ VEGF, ↓ MMP-9, ↓ IL-8 angiogenic factors	0–20 μM	[113]
Gallbladder	GBC-SD and NOZ	Anti-proliferative	↓ MMP2, ↓ MMP9, ↓ Stat3 and Akt activation, ↓ mRNA levels of MMP2 and MMP9	0–30 µM	[104]
Colon	HT-29	Inhibited cell invasion	↑ Apoptosis, ↓ Src, ↓ ERK, ↓ Akt, ↓ Bcl-2, ↑ Bax	10 μM	[54]
	HT-29 and HCT-116	Inhibiting growth of cancer cells	↑ Apoptosis, ↑ p-ERK1/2	IC_50_ of 3.2–21.4 μM,	[53]
	HT-29 cells	Anti-proliferative activities	↑ Apoptosis, ↓ HIF-1α, ↓ mPGES-1, ↓ CXCR4, ↓ VEGF, ↑ caspase 3, ↓ MMP-2, ↑ MMP-9,	0–25 µM	[52]
Prostate	DU145	Inhibit cell proliferation	↑ Apoptosis, inhibition of STAT-3 signaling pathway, ↓ p-STAT-3, ↓ IL-6, ↓ uPA, ↓ MMP-9, ↓ VEGF,	0, 10, 25 µM	[109]
	LNCaP, C4-2B and PC3	Inhibited cell growth	↓ NF-κB signaling pathway	0–25 µM	[59]

Bcl-2 associated X, apoptosis regulator (Bax), B-cell lymphoma 2 (Bcl-2), TGF-β–activated kinase 1 (TAK1), signal transducer and activator of transcription 3 (STAT3), Janus kinase 1/2 (JAK1/2), Nuclear factor kappa B (NF-κB), signal transducer and activator of transcription 5A (STAT5A), phosphoinositide 3-kinase (PI3K), AKT serine/threonine kinase (Akt), microRNA (miRNA), Cyclooxygenase-2 (COX-2), vascular endothelial growth factor (VEGF), CREB binding protein (CBP), transforming growth factor beta 1 (TGF-β1), extracellular signal regulated kinases 1/2 (ERK1/2), histone acetyltransferase (TIP60), histone lysine N-methyltransferase SUV420H2 (SUV420H2), Zinc finger E-box binding homeobox 1 (ZEB-1), Zinc finger E-box binding homeobox 2 ((ZEB-2), Urokinase-type plasminogen activator (uPA), interleukin-6 (IL-6), matrix metalloproteinase-9 (MMP-9), matrix metalloproteinase-2 (MMP-2), B-cell lymphoma-extra-large (Bcl-XL), tumor necrosis factor alpha (TNF-α), Bcl-2 associated agonist of cell death (Bad), reactive oxygen species (ROS), microsomal prostaglandin E synthase-1 (mPGES-1), C-X-C chemokine receptor type 4 (CXCR4), hypoxia inducible factor-1 alpha (HIF-1α), interleukin-8 (IL-8), Poly(ADP-ribose) polymerase (PARP), ATP binding cassette subfamily G member 2 (ABCG2), octamer binding transcription factor 4 (Oct4), Mcl1 apoptosis regulator, Bcl2 family member (Mcl-1), enhancer of zeste 2 polycomb repressive complex 2 subunit (EZH2), GLI family zinc finger 1 (Gli-1), inducible nitric oxide synthase (iNOS), lipopolysaccharide (LPS), truncated BH3 interacting domain death agonist (tBid), aldehyde dehydrogenase 1 family member A1 (ALDH1A1), DNA damage inducible transcript 3 (DDIT3), growth arrest and DNA damage inducible gene 153 (GADD153), CCAAT enhancer binding protein beta (C/EBPβ).

**Table 2:** Anticancer effects of garcinol based on in vivo studies.

Type of Cancer	Animal Models	Effects	Mechanisms	Dose	Duration	Ref.
Skin (Melanoma)	Male Balb/c mice injected subcutaneously with B16F10 tumor	Tumor inhibition	↑ Metastasis, ↑ Apoptosis, ↑ Bax and ↓ Bcl-2. ↑ Caspase-3, ↑ Caspase-9	25 mg/kg	20 days	[85]
Head	Male athymic nu/nu mice inoculated subcutaneously with CAL27 cells	Inhibited tumor growth	↓ constitutively activated STAT3, ↓ c-Src, JAK1/2, ↓ NF-κB, ↓ TAK1	1–2 mg/kg	4 weeks	[103]
Brain (Glioblastoma)	NOD/SCID mice inoculated subcutaneously with U87MG cells	Inhibited tumor growth	↑ STAT3 and ↑ STAT5A, ↑ hsa-miR-181d/STAT3, ↑ hsa-miR-181d/5A ratio, ↑ Bax, ↓ Bcl-2	1 mg/Kg	4 weeks	[106]
Cervical	Male BALB/c nu/nu mice5 inoculated subcutaneously with Hela cells	Inhibited tumor growth	↑ Apoptosis, ↑ T-cadherin knockdown of T-cahderin, ↑ P13K/AKT signaling pathway	1 mg/kg and 2 mg/kg	5 weeks	[42]
Oral (tongue)	Male F344 rats	Reduction in cell proliferation	↓ 4-NQO-induced tongue neoplasms, ↓ BrdU-labeling index, cyclin D1-positive cell ratio	100 ppm or 500 ppm	32 weeks	[114]
Esophageal	Male BALC/c nude mice Intravenously injected with KYSE150 cells via the tail vein	Reduced tumorincidence	↓ p300 and TGF-β1 signaling pathways, ↓ protein levels of p300/CBP (transcriptional cofactors and HATs), ↓ p-Smad2/3 expression in the nucleus	20 mg/kg	5 weeks	[105]
Breast	Female homozygous ICR SCID mice inoculated subcutaneously with MDA-MB-231	Inhibited tumor growth	↓ STAT-3 signaling pathway, ↓ p-STAT-3, ↓ IL-6-induced STAT-3 signaling, ↓ VEGF ↓ MMP-9	5 mg/day	4 weeks	[109]
	Female homozygous ICR SCID mice inoculated subcutaneously with MDA-MB-231	Inhibited tumor growth	↓ NF-kB, ↓ miRNAs, vimentin, ↓ β-catenin, miR-200s, ↓ let-7s, ↓ NF-κB, ↓ Wnt signaling pathways.	5mg/d/animal	4 weeks	[48]
	Male Balb/c mice inoculated with mammary carcinoma 4T1 cells	Antitumor anti-metastasis effects	↓ caspase-3, ↓ cytosolic Ca^2^+-iPLA2) ↓ NF-κB, ↓ Twist1	1 mg/ kg	5 weeks	[21]
Lung	Female NMRI (nu/nu) mice inoculated subcutaneously with A549 cells	Inhibited tumor growth	↓ALDH1A1, ↑DDIT3	15mg/kg	40 day	[110]
	NOD/SCID mouse bearing H441 tumor sphere	Inhibited tumor growth	↓ Wnt/β-catenin/STAT3 axis, ↓ p-LRP6, ↓ Axin2, ↓ β-catenin, ↓ cyclin D1	5 mg/kg	9 weeks	[50]
Hepatocellular	Female athymic nu/nu mice inoculated subcutaneously PLC/PRF5 cells	Inhibited tumor growth	↑ Apoptosis, ↓ IL-6 induced STAT3 activation, ↓ STAT3 acetylation, ↓ cyclin D1, ↓ Bcl-2, ↓Bcl-xL, ↓ survivin, ↓ Mcl-1, ↓VEGF	1mg/kg and 2 kg/kg	3 weeks	[60]
Pancreatic	Male KPC mice (K-rasLSL.G12D/+; p53R172H/+; PdxCretg/+)	Reduction in tumor volumes	↓ COX2, ↓ cyclin D1, ↓ VEGF, ↓ Wnt/b-catenin	0.5 g/kg	5 weeks	[115]
Colon	Male F344 rats	Inhibition of aberrant crypt foci	↓ PCNA index, ↓ NO and 02, ↓ iNOS, ↓ COX-2	15 mg/kg	5 weeks	[83]

Bcl-2 associated X, apoptosis regulator (Bax), B-cell lymphoma 2 (Bcl-2), TGF-β–activated kinase 1 (TAK1), signal transducer and activator of transcription 3 (STAT3), Janus kinase 1/2 (JAK1/2), nuclear factor kappa B (NF-κB), low density lipoprotein receptor-related protein 6 (LRP6), twist-related protein 1 (TWIST1), histone acetyl transferase (HAT), signal transducer and activator of transcription 5A (STAT5A), proliferating cell nuclear antigen (PCNA), phosphoinositide 3-kinase (PI3K), AKT serine/threonine kinase (Akt), microRNA (miRNA), cyclooxygenase-2 (COX-2), vascular endothelial growth factor (VEGF), wingless (Wnt), independent phospholipase A2 (iPLA2), CREB binding protein (CBP), transforming growth factor beta 1 (TGF-β1), interleukin-6 (IL-6), Matrix metalloproteinase-9 (MMP-9), B-cell lymphoma-extra-large (Bcl-XL), Mcl1 apoptosis regulator, Bcl2 family member (Mcl-1), inducible nitric oxide synthase (iNOS), aldehyde dehydrogenase 1 family member A1 (ALDH1A1), DNA damage inducible transcript 3 (DDIT3).

## Abbreviations

Extracellular signal regulated kinases 1/2 (ERK1/2), Phosphoinositide 3-kinase (PI3K), AKT serine/threonine kinase (Akt), Wingless (Wnt), Signal transducer and activator of transcription 3 (STAT3), Nuclear factor kappa B (NF-κB), Platelet-derived growth factor receptors (PDGFRs), Oral squamous cell carcinoma (OSCC), Cyclooxygenase-2 (COX-2), B-cell lymphoma-extra-large (Bcl-XL), Bcl-2 associated X, apoptosis regulator (Bax), p300/CBP associated factor (PCAF), Alteration/Deficiency in Activation 3 (ADA3), Receptor tyrosine kinase (RTK), Proliferation cell nuclear antigen (PCNA), Poly(ADP-ribose) polymerase (PARP), NADPH oxidase 1 (NOX1), Apoptosis signal regulated kinase 1 (ASK1), Thioredoxin 1 (Trx1), B-cell lymphoma 2 (Bcl-2), Mitogen activated protein kinase kinase 4/7 (MKK4/MKK7), c-Jun N-terminal kinase (JNK), Stress activated protein kinase (SAPK), Epithelial-to-mesenchymal (EMT), Low density lipoprotein receptor-related protein 6 (LRP6), Disheveled segment polarity protein 2 (Dvl2), Liver cancer stem cell (LCSC), Cyclin dependent kinases (CDK), Mitogen activated kinase like protein (MAPK), Microsomal prostaglandin E synthase-1 (mPGES-1), Prostaglandin E synthase 2 (PGE2), Hypoxia inducible factor-1 alpha (HIF-1α), C-X-C chemokine receptor type 4 (CXCR4), Vascular endothelial growth factor (VEGF), Matrix metalloproteinase-2 (MMP-2), Matrix metalloproteinase-9 (MMP-9), Cytochrome c (cyt c), Phosphorylated mechanistic target of rapamycin kinase (p-mTOR) pathway, Hepatocellular carcinoma (HCC), Src homology 2 (SH2), Reactive nitrogen species (RNS), Reactive oxygen species (ROS), Inducible nitric oxide synthase (iNOS), 1,1-diphenyl-2-picrylhydrazyl (DPPH), Lipopolysaccharide (LPS), Histone acetyltransferase (HAT), Head and neck carcinoma (HNSCC), Janus kinase 1/2 (JAK1/2), TGF-β-activated kinase 1 (TAK1), Inhibitor of IκB kinase (IKK), Inteleukin-6 (IL-6), Independent phospholipase A2 (iPLA_2_), Twist-related protein 1 (Twist1), Inteleukin-6 (IL-8), Gallbladder carcinoma cells (GBC), Transforming growth factor beta 1 (TGF-β1), CREB binding protein (CBP), Pancreatic cancer (PaCa), Tumor necrosis factor related apoptosis inducing ligand (TRAIL), c-FLICE-like inhibitory protein (c-FLIP), Differential calorimetry (DSC), World health organization (WHO), Poly(lactic-co-glycolic acid) (PLGA), D-α-tocopheryl polyethylene glycol 1000 succinate (TPGS), Silver nanoparticles (AgNPs).
